# Biobridge: An Outlook on Translational Bioinks for 3D Bioprinting

**DOI:** 10.1002/advs.202103469

**Published:** 2021-12-03

**Authors:** Yawei Gu, Aurelien Forget, V. Prasad Shastri

**Affiliations:** ^1^ Institute for Macromolecular Chemistry University of Freiburg Freiburg 79104 Germany; ^2^ Bioss‐Centre for Biological Signalling Studies University of Freiburg Breisgau 79104 Germany

**Keywords:** immunomodulation, instructive bioinks, organotypic vasculature, proregenerative bioinks, standardization

## Abstract

3D‐bioprinting (3DBP) possesses several elements necessary to overcome the deficiencies of conventional tissue engineering, such as defining tissue shape a priori, and serves as a bridge to clinical translation. This transformative potential of 3DBP hinges on the development of the next generation of bioinks that possess attributes for clinical use. Toward this end, in addition to physicochemical characteristics essential for printing, bioinks need to possess proregenerative attributes, while enabling printing of stable structures with a defined biological function that survives implantation and evolves in vivo into functional tissue. With a focus on bioinks for extrusion‐based bioprinting, this perspective review advocates a rigorous biology‐based approach to engineering bioinks, emphasizing efficiency, reproducibility, and a streamlined translation process that places the clinical endpoint front and center. A blueprint for engineering the next generation of bioinks that satisfy the aforementioned performance criteria for various translational levels (TRL1‐5) and a characterization tool kit is presented.

## Graphical abstract

3D‐bioprinting (3DBP) is a promising technology that serves as a bridge to clinical translation. The translational potential of 3DBP depends on the development of the next generation of bioinks. Translational bioinks need to possess proregenerative attributes, while enabling printing of stable structures that survive implantation and evolve into functional tissue.

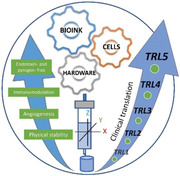

## 1 Introduction

### 1.1 Bioinks—the Present and Future

Merriam‐Webster dictionary defines ink as “a colored material for writing and printing,” where the attributes of the ink lie in the properties of the colloids. Drawing an analogy, in the broadest sense a bioink can be viewed as a medium to deposit biological information in a well‐defined pattern. The current definition of bioink as proposed by the International Society for Biofabrication is “a formulation of cells suitable for processing by an automated biofabrication technology that may also contain biologically active components and biomaterials.”^[^ ^1^ ^]^ This wide definition certainly takes a variety of conditions into consideration, such as bioprinting cell aggregates that can serve as both the source of the biology and the source of the scaffolding.^[^ ^2^, ^3^ ^]^ However, in order to realize functional tissues in vivo one has to combine structure and function, and this cannot be realized purely through implantation of a cell‐biomaterial construct. Thus, pivoting from the current definition, here we propose bioink as a composite of biomaterial medium and biological information (e.g., cells, pharmaceutical molecules, growth factors) that can invoke a predictable biological response. In essence this revised definition combines the attribute of a carrier of cells (or biomolecules) with that of an instructive biomaterial. Departing from the current definition, in this expanded definition, bioink in the broadest sense is a material that can be formed into complex structure through extrusion, is capable of invoking biological response, which can either be controlling a biological response or supporting vascularization, and/or providing supportive environment for tissues to remodel. Several studies have shown that a hydrogel by itself can trigger a regenerative process and lead to functional tissue formation through endogenous recruitment of resident cell population.^[^ ^4^, ^5^, ^6^ ^]^ Therefore, a bioink per se does not necessarily need to carry cells. However, in many instances, when there is no endogenous population of cells that can be recruited, it might be necessary to include a specific cell type or a mixture of cells to get the desired outcomes. As the function of a bioprinted structure could be to support the existing tissue or to promote an existing process in a tissue, or to bridge a process between the tissues, we place emphasis on the role of the bioink in driving in vivo outcomes. Here we would envision the bioink medium as a blank canvas which can be manipulated to possess biological information and nuanced physiochemical properties. Therefore, a bioink is designed to function as a system to support cellular function and organization. or trigger communication between various cellular compartments in vivo , and not simply to deliver the cell/biological molecules.

There is substantial evidence that biomaterials are capable of invoking and controlling biological processes.^[^ ^4^, ^7^, ^8^, ^9^, ^10^ ^]^ The idea of biomaterials as instructive environments was first put forth by Jefferey Hubbell in the early 2000s. An instructive biomaterial is defined as a material that can modulate cell behavior through its mechanical, chemical, physical or biological properties, to invoke developmental processes leading to morphogenesis.^[^ ^7^ ^]^ Biomaterial features such as scaffold porosity,^[^ ^11^ ^]^ nanoscale topography,^[^ ^8^ ^]^ polymer surface charge,^[^ ^12^ ^]^ and tethered peptides (such as RGD)^[^ ^4^ ^]^ or proteins (such as integrin, INF‐*γ*)^[^ ^13^, ^14^ ^]^ have been shown to direct cell fate and function. Several synthetic nondegradable (e.g., Pluronic and polyethylene glycol), and degradable polymers (e.g., poly(*ε*‐caprolactone), and poly(lactic acid)), and polymers sourced from flora (e.g., nanocellulose, alginate, carrageenan, and agarose) and fauna (e.g., collagen, gelatin, hyaluronic acid (HA))^[^ ^15^ ^]^ have been explored in 3D‐printing. However, when it comes to printing cells, systems capable of yielding hydrogels such as polysaccharides, proteins, and peptides are uniquely suited as bioinks, as they possess many of the properties of the extracellular matrix (ECM), can be easily engineered to dictate specific cellular function and processed in aqueous medium. When combined with matrix mechanics, hydrogel‐based bioinks can serve as an authentic equivalent to tissue environments found in both embryo and adult mammals, and can serve as a reproducible set of physicochemical cues for driving tissue organization.^[^ ^7^, ^16^ ^]^ However, the challenge here lies in developing hydrogel‐based systems that yield predictable outcomes, have a clinical translational potential, and yet be compatible with various extrusion‐based bioprinting (EBB) platforms.

### 1.2 Considerations for Developing Bioinks for Clinical Translation

**Figure 1:**
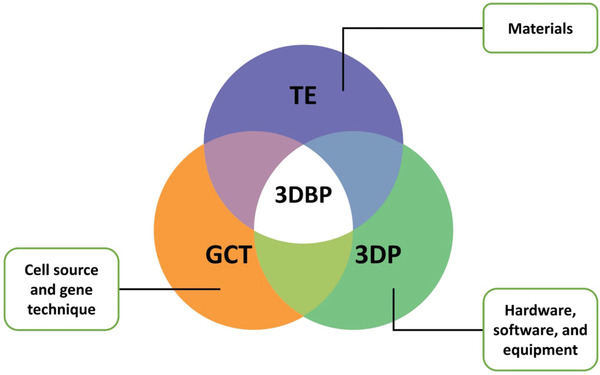
3DBP—a convergence of technologies: Overlapping elements between tissue engineering (TE), 3D printing (3DP), gene & cell therapy (GCT), and 3D bioprinting (3DBP).

3DBP as a technology has developed rapidly over the past decade and is being explored in regenerative medicine, drug delivery, disease modeling (organ‐on‐a‐chip), and drug screening.^[^ ^17^, ^18^, ^19^ ^]^ In this article, we focus on the use of 3DBP in regenerative medicine. 3DBP and tissue engineering (TE) share many commonalities, as both serve as fabrication method to augment, restore, or repair tissue. Both, hold much promise and possess the potential to be transformational, depend on access to cells that can be manipulated into tissues, and require biomaterials that promote tissue morphogenesis. But unlike TE, in 3DBP the ability to evolve (print) structures of complex shapes through direct deposition of cells should in theory enable rapid evolution of new construct paradigms, and thus a nimbler technology platform. By combining conventional 3D printing or EBB with highly specialized bioinks, models of the alveolus (lung sac)‐like structure and heart with anatomically accurate chambers have been printed.^[^ ^20^, ^21^ ^]^ 3DBP can benefit from the successes and missteps of TE.^[^ ^22^ ^]^ Tissue engineering had to grapple with the absence of standardization and lack of early bench‐to‐bedside success, which led to a slow development phase in the new millennium after an initial vigorous progress.^[^ ^23^ ^]^ The logistical challenges of validating the procurement, processing, storage of human cells and the costs associated with meeting stringent regulatory and ISO (International Organization for Standardization) standards have significantly limited the number of tissue‐engineered medicinal products available in market.^[^ ^24^, ^25^ ^]^ 3DBP confronts similar challenges and has many overlapping elements with TE as illustrated in **Figure** **1**. Therefore, the regulations for gene & cell therapies, and the current standard for preclinical testing of implantable medical devices (ISO 10993‐1:2018),^[^ ^26^ ^]^ and additionally, the current legislation and regulations on tissue‐engineered medical products in different countries which has been summed up in a recent review^[^ ^25^ ^]^ should provide valuable references for the development of implantable clinical products based on 3DBP. From the perspective of scientists, on one hand we need to familiarize ourselves with regulatory standards to make “scholarly acceptable” outcomes also “clinically acceptable,” and on the other hand we need to make ourselves aware of scientific and clinical requirements to ensure 3DPB‐based products can enter the clinical translation pipeline. Thus, for 3DBP to evolve into a sustainable and vibrant multidisciplinary field, a framework for evolving laboratory discoveries into viable clinical solutions needs to be developed. A crucial element in realizing the latent potential of 3DBP therefore is a portfolio of bioinks that are clinically acceptable, have proven performance and biocompatibility, can be adopted by various printing ecosystems, and is suitable for preclinical screening and clinical implementation.

### 1.3 Property Profile and Tool Kit for the Development of Translatable Bioinks

**Figure 2:**
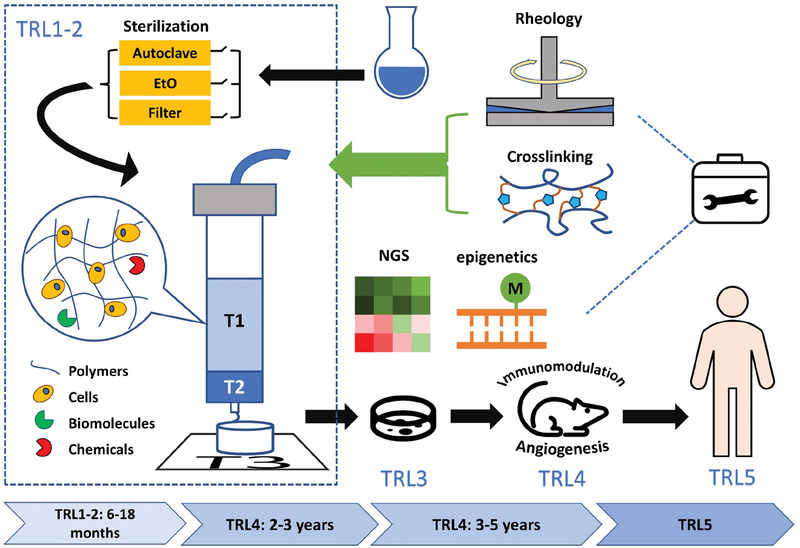
Scheme for development of translatable bioinks. The tool kit includes rheological tests and crosslinking strategies, which are necessary in phases TRL1 and TRL2, including sterilization, optimization of bioink formulations, printing process simulation and analysis, printing, and post‐printing characterization that has direct bearing on TRL3‐TRL5. In TRL3, the incorporation of next‐generation sequencing (NGS) for example, RNAseq and epigenetics will be critical to ascertain the effect of the bioink and processing on cell phenotype and identifying factors that can promote pro‐regenerative immune responses upon implantation (T1, T2, and T3 denote cartridge temperature, nozzle temperature, and printing platform temperature). In TRL4, bioink‐cell systems are validated in animal disease models that are comparable to diseases targeted in TRL5 and using good laboratory practices (GLP) compliant workflow to ensure that the data can meet regulatory requirements.

**Figure 3:**
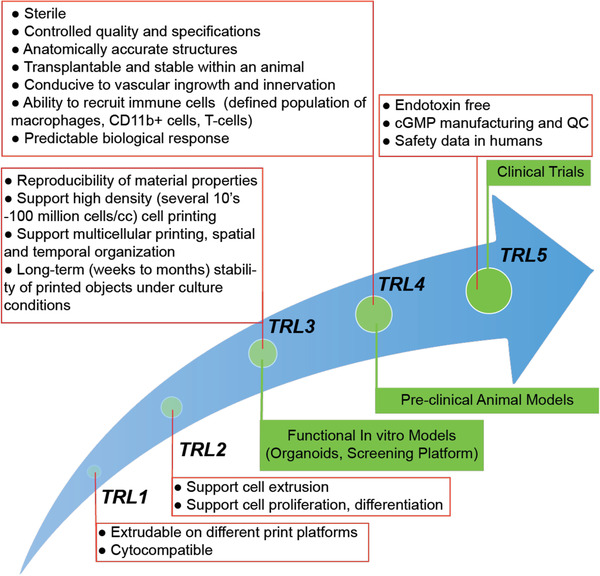
Bioinks translational path to the clinic. The red boxes list properties essential for that translational level in addition to properties identified for previous translational levels. Green boxes denote the key applications or activity associated with that translational level.

In order to transform bioprinted structures into viable tissue, printed structures should preserve their shape during culture, be mechanically robust to be handled and surgically implanted and survive within an organism for a certain period of time depending on its intended function and the site of transplantation. This requires standardization of bioink properties. The metrics for such a standardization have to be based on outcomes from studies using 3D‐bioprinted systems that are targeted towards a defined clinical application. Therefore, bioinks should possess the **THREE P's**: predictable *physical properties*, predictable *biological response*, and predictable *in vivo outcomes*. Based on this reasoning we define translational levels (TRL1‐TRL5) and associate them with various stages of development of 3DBP solution (**Figure** **2**). To accomplish a collaborative ecosystem that can lead to translational outcomes, bioink property prolife needs to be standardized using a performance criteria and characterization tool kit against a TRL (**Figure** **3**). The objective of this review is to provoke a robust discussion between all stakeholders in the bioprinting field to drive a consensus framework and foster early interactions between industry and academic laboratories to realize some of the objectives presented herein.

## 2 Bioink Property Profile

### 2.1 Thermal Characteristics of Bioinks

Unlike fused deposition modeling (fused filament fabrication), in bioprinting, as the bioink contains temperature‐sensitive components such as cells, proteins, or cytokines, the ink cartridge temperature (holding temperature) and printing temperature (at needle/needle tip) must be appreciably lower. Since a majority of studies with bioinks have involved cells, physiological temperature is considered to be a ideal setting for bioprinting. Because hyperthermia has a direct impact on plasma and mitochondrial membrane stability, protein synthesis, and cell function, high temperature should be avoided when possible.^[^ ^27^ ^]^ Prolonged exposure of cells to elevated temperatures while not immediately fatal to cells, can lead to increased intracellular calcium and sodium levels,^[^ ^28^ ^]^ elevated mitochondrial membrane potential, and induce reactive oxygen species (ROS) production in cells which can be detrimental.^[^ ^29^, ^30^ ^]^ Interestingly, nuclear matrix (and material) shows significant changes at 40 °C (high grade fever). In humans, at and above 41 °C, direct cell death has been observed with increasing cell death even with minute changes increases in temperature. Therefore, the range of holding and printing temperatures of bioink needs to be quite narrow and ideally not exceed 39 °C, which corresponds to a low‐grade fever.^[^ ^31^ ^]^ Although a few reports mention loss in cell viability during bioprinting at temperatures below physiological values,^[^ ^32^, ^33^ ^]^ whether these nonphysiological temperatures would induce long‐term side effects needs further investigation. In thermally responsive bioinks, viscosity is a temperature‐dependent variable and therefore, shear‐stress experienced by cells can be vastly different as they traverse the various elements of printing. Hence, the bioink operating temperature needs to be between ambient temperature (laboratory or clinical environmental setting) and physiological temperature and be the first criteria for material selection. Based on this temperature range, formulations of bioinks could be screened as a function of viscosity, with the objective of identifying the minimum viscosity for printability, which is discussed later. Interestingly, this restriction can be advantageous as it dramatically narrows the choice of biomaterials, and the chemistry that can be leveraged during printing.

### 2.2 Medical Grade Bioinks

From a translation standpoint, it is necessary that bioinks are sterile, free of endotoxins, and lack pyrogenicity. Pyrogenicity can be caused by bacterial debris, denatured proteins, and leachants. Commercially sourced hydrogel‐forming materials of bacterial or biological origin such as HA, gelatin, and alginate often contain high levels of endotoxins.^[^ ^34^, ^35^ ^]^ Therefore, extensive purification steps are necessary to yield medical‐grade materials for formulation into bioinks. It has been shown that HA and HA fragments with low‐no endotoxin burden do not stimulate macrophages and dendritic cells in inflammatory models.^[^ ^36^ ^]^ Although a type of gelatin with low endotoxin burden (Rousselot x‐Pure)^[^ ^37^ ^]^ has been available since 2018, gelatin‐based bioinks (e.g., gelatin‐methacrylate (GelMA)) are chemically modified and this imports two sets of issues, namely, changes in endotoxin burden, and purity and reproducibility of the chemical composition of the modified gelatin. In bioinks that require postpolymerization (chemical or light), the toxicity of substances in the reaction should be taken into consideration. While curable monomers are rarely used in 3DBP with possible reasons being that systems comprising purely of a photopolymerizable monomer usually cannot reach the required viscosity; in systems containing cross‐linkable moieties, extensive purification is essential to remove unreacted components, as they are often toxic, and can leach out over an extended period from the hydrogel.^[^ ^38^ ^]^ Nonetheless, efforts in tissue‐engineering have already screened several candidates for hydrogels (polyethylene glycol (PEG),^[^ ^39^, ^40^ ^]^ HA,^[^ ^41^, ^42^ ^]^ gellan gum^[^ ^43^ ^]^), with some of these materials in use in therapeutics approved by the FDA (United States Food and Drug Administration). The stabilization of a bioink formulation using temperature‐responsive biopolymers such as gelatin and gellan to facilitate postprocessing of acrylate‐based bioink formulations has been explored.^[^ ^43^, ^44^ ^]^ Nevertheless, leachables (residual crosslinker(s) and photoinitiator) remain the Achilles heel of photocurable bioinks such as GelMA, PEG‐diacrylate, and collagen‐methacrylate (ColMA). Therefore, defining residual levels that are clinically acceptable, protocols for their removal and the reliability of the purification process will become key factors in determining the fate of these and other bioinks based on similar chemistries in clinical application.

Another aspect very critical for translation although not often studied is how to sterilize bioinks, and whether the FDA‐approved terminal sterilization methods would change the property of bioinks. A recent study compared the effects of three sterilization methods (autoclaving, sterilization by filtration through membrane with a pore size of 0.22 µm, and ethylene oxide (EtO) sterilization) on most widely used bioinks and concluded that EtO sterilization was a relatively safer and less destructive method. As many of the bioinks for 3DBP possess high viscosity, filtration in not practical. High pressure and temperature encountered during autoclaving could severely degrade protein‐ or peptide‐based materials like gelatin, destroying their molecular structure and altering their rheological behavior.^[^ ^45^ ^]^ In another study on GelMA, the most widely used bioink, the FDA‐approved terminal sterilization methods were found to influence key properties of GelMA namely, mechanical properties, gelling behavior, and cell–material interaction to varying degrees.^[^ ^46^ ^]^ Thus, the choice of the sterilization will also influence the determination of the initial bioink‐formulations and this needs to be defined prior to commencing an exhaustive pre‐clinical study.

### 2.3 Shear‐Thinning, Predictable Gelation, Fidelity, and Mechanical Stability

One of the challenges in 3DBP as we scale up from small rodent models to large mammals and eventually humans, is the need to print large functional structures. Printing large high‐aspect‐ratio structures require bioinks that exhibit structural integrity and have mechanical properties that can support the printed structure. One approach to realizing high‐aspect‐ratio and complex structures is the use of fugitive phase such as Pluronic^[^ ^47^ ^]^ and gelatin microparticle slurry as in the freeform reversible embedding of suspended hydrogels (FRESH) process^[^ ^48^ ^]^ followed by postprocessing of the printed structures using, for example, photopolymerization. Another strategy to modify the viscosity of the bioink is adding viscosity modifiers to promote stability of printed structures followed by postprocessing using crosslinking chemistries. The advances in past five years in 3DBP of cell‐laden bioinks and the structures realized using these bioinks are summarized in **Table** **1**. One important aspect to consider in the processing of printed structures during or after printing is loss of fidelity and delamination of printed layers. When materials undergo sol‐gel transition, shrinkage of the gel phase can result in mismatch between the prescribed g‐code and the structure being printed. This can lead not only to discontinuity in printed objects, but also limit the height that can be printed and impact the reproducibility of printed structures. These challenges can be further compounded when cells are present.

**Table 1:** Summary of advances in 3D bioprinting with different cell‐laden bioinks in past five years

	Polymer system	Bioactive (A) or bioinert (I)	Printing method	Free‐standing print	Printed geometries	Rheological modifiers	Supporting or fugitive phases	Crosslinking mechanism: physical (P) or chemical (C)	Refs.
	Nature‐derived polymers								
Agarose	Native agarose	I	Extrusion	Yes	Grid and hollow cylinder	Yes, laponite nanosilicates	No	P/temperature induced phase transition	^[^ ^54^ ^]^
	Native agarose	I	Extrusion	^a)^	Honeycomb‐like lines	Yes, alginate	No	Non‐covalent C/Ionic crosslinking with calcium (Ca)	^[^ ^55^ ^]^
	Carboxylated agarose (CA)	I	Microvalve (drop‐on‐demand)	Yes	Cylinder	No	No	P/temperature induced phase transition	^[^ ^56^ ^]^
	CA/native agarose	I	Extrusion	Yes	Hollow tube, S‐shape hollow tube, bifurcated hollow tube, hemisphere, and hollow tube with cross	No	No	P/temperature induced phase transition	^[^ ^57^ ^]^
Alginate	Alginate	I	Extrusion	No	Honeycomb‐like construct	No	Yes, agarose slurry	Non‐covalent C/Ionic crosslinking with Ca	^[^ ^58^ ^]^
	Alginate	I	Extrusion	Yes	3D human ear and nose model	Yes, pluronic F127	Yes, pluronic F127	Non‐covalent C/Ionic crosslinking with Ca	^[^ ^59^ ^]^
	Alginate sulfate	I	Microvalve	Yes	3D human ear model	Yes, nanocellulose	No	Non‐covalent C/Ionic crosslinking with Ca	^[^ ^60^ ^]^
Collagen	Collagen	A	Extrusion	No	^b)^3D tri‐leaflet heart valve model, and 3D human heart model	No	Yes, gelatin slurry	C/glutaraldehyde	^[^ ^21^ ^]^
	Collagen	A	Extrusion	Yes	Latticed cube	Yes, alginate	No	C/glutaraldehyde	^[^ ^61^ ^]^
	ColMA	A	Extrusion	No	3D human corneal model	No	Yes, gelatin slurry and plastic support	C/UV polymerization	^[^ ^62^ ^]^
	Functionalized rh‐*h‐*collagen^c)^	A	Two‐photon polymerization	^a)^	Letters and logo	No	No	C/UV polymerization	^[^ ^63^ ^]^
Chitosan	Chitosan	I	Extrusion	No	Latticed cube	No	Yes, polycaprolactone	P/Temperature induced phase transition	^[^ ^64^ ^]^
	Carboxymethyl chitosan	I	Extrusion	Yes	Square grid	Yes, agarose and alginate	No	Non‐covalent C/Ionic crosslinking with Ca	^[^ ^65^ ^]^
Decellulari‐zed matrix (DM)	Adipose tissue	A	Extrusion	No	Grid of alternating PCL and DM in a cube geometry	No	Yes, PCL	P/Temperature induced phase transition	^[^ ^66^ ^]^
Gelatin	Carbohydrazide‐modified gelatin	A	Extrusion	No	Crosshatched grid, meshed tubes, meshed spheres, ball‐in‐a‐cage construct, and humerus model	No	Yes, gelatin microparticles suspended in oxidized alginate	C/Schiff base formation	^[^ ^67^ ^]^
	GelMA	A	Extrusion	No	Cube with hollow tube, grid, and hollow drum	No	Yes, carbopol solution	C/UV polymerization	^[^ ^68^ ^]^
Gellan gum	Gellan gum/poly (ethylene glycol) diacrylate (PEGDA)	I	Extrusion	Yes	Hollow cylinder, and pentagram‐shaped tube	No	Yes, crosslinked PEGDA	C/UV polymerization	^[^ ^69^ ^]^
Hyaluronic acid	HA	A	Extrusion	Yes	Cylinder	Yes, alginate	No	Non‐covalent C/Ionic crosslinking with Ca	^[^ ^70^ ^]^
	HA‐hydroxyethyl acrylate‐GelMA	A	Extrusion	^a)^	Grid	No	No	C/UV polymerization	^[^ ^71^ ^]^
	HA‐methacrylate (in the form of microstrands)	A	Extrusion	Yes	3D human ear model	No	No	C/UV polymerization	^[^ ^72^ ^]^
Fibrin	Fibrinogen	A	Extrusion	Yes	Object with overhanging structures, 3D human ear model	Yes, alginate and gelatin	No	P+ noncovalent C/Temperature induced phase transition combining with ionic crosslinking with Ca	^[^ ^73^ ^]^
Matrigel	Matrigel	A	Extrusion	Yes	Hollow cylinder, star‐, triangle‐, and square‐shaped constructs	Yes, agarose	No	P/temperature induced phase transition	^[^ ^74^ ^]^
Silk	Silk/PEG	A	Extrusion	Yes	Latticed cylinder, latticed cube, and 3D ear model	No	No	P/temperature induced phase transition	^[^ ^75^ ^]^
	Synthetic polymers								
PEG	PEG‐norbornene (in form of microgels)	I	Extrusion	Yes	Honeycomb‐like construct, and hollow cylinder	No	No	C/UV polymerization	^[^ ^76^ ^]^
	PEGDA	I	Extrusion	Yes	Hollow cylinder, pentagram‐shaped tube, sharp cone structure, reverse square prism, cube, 3D human ear model, and 3D human nose model	Yes, gellan gum	No	C/UV polymerization	^[^ ^69^ ^]^

^a)^ Refers to 2D structures lacking any appreciable height;

^b)^ In this case the cells and collagen were printed separately;

^c)^ Recombinant protein based on human collagen type I, functionalized with methacrylamide, norbornene, or thiol functionalities.

During bioprinting the bioink goes through three major processes: I) shear‐induced extrusion through a narrow tube, II) transient assumption of geometry, and III) long‐term preservation of geometry. The second and the third processes are two distinct steps in cases involving post‐processing, while in cases like in‐line crosslinking, these two processes would occur simultaneously. However, we would still like to delineate them as two phases, as the mechanisms involved in structure formation might be different for structure preservation. Therefore, for the three processes mentioned above shear thinning, predictable gelation, and mechanical stability, respectively, are properties we deem to be critical. During bioprinting, the bioink undergoes a transition from a transformable state (the first process) to a stable state (the third process). During extrusion, shear‐thinning is an essential in a bioink, as the bioink must undergo a holding‐flowing‐sizing process, indicating two viscous phases (holding and sizing), and a flexible phase in between. The shear‐thinning property of bioinks can also significantly diminish shear‐induced stress on cells, as the higher shear stress the cells endure the more likely they will be damaged.^[^ ^49^ ^]^ During the deposition of the bioink predictable gelation behavior can guarantee a dynamic balance between bioink flow and geometry stability, generating structure integrity, while avoiding delamination between layers. Cell‐laden bioinks need to be extensively characterized for their rheological behavior in the various aforementioned process steps to ensure homogeneity of the print. Recapitulating stages in embryonic development will necessitate bioinks that have high loading of cells (several 10s to 100 million cells per mL) to ensure cell–cell contact. Since a cell is elastic, deformable, uncompressible object, during print one could expect phase separation and at high volumetric cell densities, due to large cell volume fraction, disruption of gel networks can also occur.^[^ ^50^ ^]^ Furthermore, when cells are above percolation threshold, during extrusion‐based bioprinting, compaction (gravity‐induced settling and shear induced) can result in rheological properties that exhibit time‐dependent heterogeneities leading to anomalies such as inhomogeneous distribution of cells in printed structures.^[^ ^51^, ^52^, ^53^ ^]^ After printing, the bioink should possess the capacity to support its own weight, exhibit stable geometry that can withstand in vitro culture and in vivo implantation, while maintaining the fidelity as much as possible.

## 3 Bioinks Promoting Regeneration

In the development of bioinks with proregenerative attributes, one has to consider the host biology and the biomaterial properties. In this regard, the immunological response to the biomaterial has an important role and this aspect maybe be associated with (or correlated to) the physicochemical properties of the bioink.

### 3.1 Immunomodulation versus Angiogenesis

**Figure 4:**
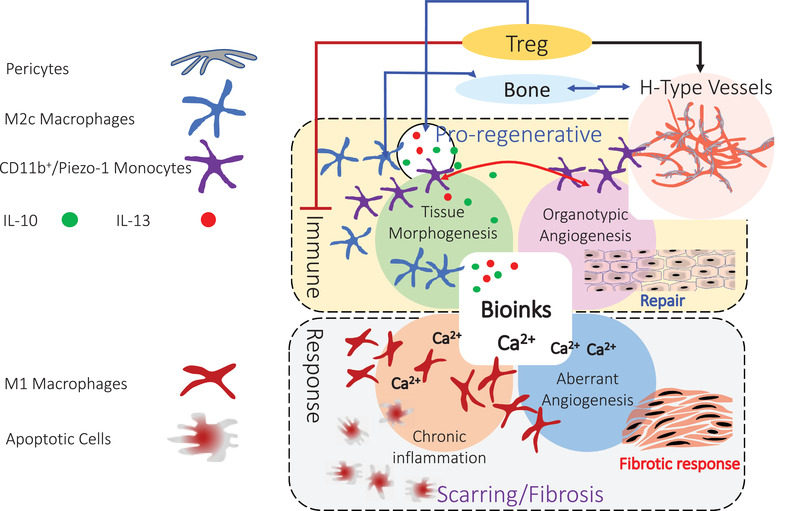
Biological events promoting regeneration/repair versus scarring. The biological response to a bioink is a critical aspect of the translational process. Bioinks capable of inducing recruitment of immune cells such as M2c macrophages, and Treg's can drive a signaling environment that is favorable to cellular and organ homeostasis leading to healing. Functionalization of bioinks with cell adhesion motifs are necessary for endothelial cell proliferation and vascular network formation. Such nascent vascular networks can be stabilized by pericytes and mechanosensing (Piezo‐1) positive circulating monocytes. Local delivery of interleukins 10 and 13 can also aid in tissue homeostasis. In contrast, bioinks possessing pro inflammatory traits such as being a source of calcium or having motifs that activate M1 macrophages can drive a hyperinflammatory signaling environment and differentiation of fibroblasts in to myofibroblasts progressing to pathological scarring.

Toward proregenerative bioinks, two aspects are critical: I) immune response to the bioink, and II) recruitment of new blood vessels—sprouting angiogenesis (**Figure** **4**). There is gathering evidence that both the innate and adaptive immune response play a pivotal role in regenerative processes. The immunological response to an implanted bioink therefore represents an important bioink design parameter. In the innate immune response, macrophage polarization denotes an important event, with M1 macrophages providing pro‐inflammatory cues and M2 macrophages contributing to tissue repair and regeneration.^[^ ^77^, ^78^, ^79^ ^]^ Also, regulatory T‐cells (Treg) have been shown to play a role in regenerative processes.^[^ ^80^ ^]^ Interestingly, Treg promotes regenerative processes by suppressing the immune system. Treg has been shown to facilitate inflammation resolution by promoting efferocytosis (apoptotic cell clearance) by secreting interleukin‐13 (IL‐13), which in turn stimulates IL‐10 production in macrophages.^[^ ^81^ ^]^ In fact, IL‐10 has been shown to be necessary for polarization of macrophages to the M2c (proregenerative) phenotype.^[^ ^82^, ^83^ ^]^ Furthermore, tissue‐resident Treg's have been shown to play a role in bone formation through cross‐talk with bone‐forming cells via CD39‐CD73 (adenosine) adenosine receptor pathway.^[^ ^84^ ^]^ One approach to promote regenerative microenvironment is to engineer bioinks to present or deliver molecules to influence the local immune response.^[^ ^85^, ^86^ ^]^

It is now well established that vasculature is specific to a tissue (organotypic vasculature), and is critical for proper organ development and regeneration.^[^ ^87^, ^88^ ^]^ For example, it has been demonstrated that osteogenesis is coupled to the presence of Type‐H vessels (endothelial cells with high expression levels of Endomucin), and that endothelial‐notch activity promotes this coupling.^[^ ^89^ ^]^ Interestingly, it has been shown that factors that drive angiogenesis also suppress the immune system by suppressing antigen‐presenting cells and/or augmenting the anti‐inflammatory effects of Treg.^[^ ^90^ ^]^ In response to aseptic injury such as ischemia, the simultaneous activation of angiogenesis and immune suppression programs are thought to be critical in ensuring tissue homeostasis. This conclusion is borne out by the observation that polarized hematopoietic cells can support both angiogenesis and immune suppression. Such, hemostatic programs are believed to be co‐opted by tumors during their development.^[^ ^91^ ^]^ Therefore, based on this conjuncture, we propose that bioinks should inherently support angiogenesis to be biocompatible and translational.

### 3.2 Bioactiveness versus Bioinertness

**Figure 5:**
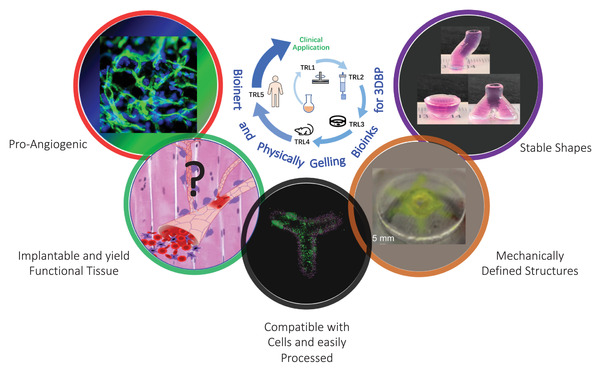
Progress in the clinical translation of inert bioinks. The key milestones in the development of inert bioinks towards clinical use stemming from efforts in our lab is summarized. Image of 'pro‐angiogenic': Reproduced with permission.^[^ ^4^ ^]^ Copyright 2019, Wiley‐VCH. Images of 'compatible with cells and easily processed' and 'machanically defined structures': Reproduced with permission.^[^ ^56^ ^]^ Copyright 2017, Wiley‐VCH. Image of 'stable shapes': Reproduced with permission.^[^ ^57^ ^]^ Copyright 2020, MDPI. Carboxylate agarose (CA)‐based bioinks such as the CANA family of bioinks, are amenable to printing on both micro extrusion and drop‐on‐demand platforms. Due to their shearing thinning properties, and decoupled sol and gel viscosity, stable, mechanically cell laden (>10^6^ cells mL^–1^) structures can be printed at physiological temperatures without the need for a support phase or post‐crosslinking or processing. Mechanically defined‐CA bioinks modified with cell adhesion sequences are capable of inducing stable angiogenesis through the recruitment of Piezo‐1+, CD11b+/CD115+ circulating monocytes. The ability of CA‐bioink based bioprinted structures to yield function tissue in vivo, however, remains to be demonstrated.

Thus far, numerous studies have focused on bioinks composed of bioactive materials, such as collagen, gelatin, and HA, as such bioactive materials are considered excellent mimics of ECM, and are efficient in attracting cell, promoting cell proliferation, or differentiation. However, whether these materials can lead to desired clinical outcomes after implantation still needs further validation, because these materials can also induce unexpected and unwanted biological activity on tissue surrounding the implantation sites, possibly resulting in “foreign body response/reaction.”^[^ ^92^ ^]^ In fact, in HA‐based dermal fillers, granuloma tissue has been frequently observed due to HA microparticles.^[^ ^93^, ^94^ ^]^ These findings in sum provide valuable impetus to explore inert biomaterials in bioprinting applications. Bioinert materials have rarely been highlighted in the development of bioinks, as they are regarded as nondegradable and therefore assumed to be nonpermissive to cells. However, natural bioactive materials like collagen, gelatin, HA, usually suffer from poor processibility, namely they are difficult to be processed into a stable shape. Bioinert materials such as agarose, carrageenan, gellan possess superior processibility and can be additionally functionalized to introduce a specific bioactive motif^[^ ^9^, ^95^, ^96^ ^]^ (Table 1). Furthermore, bioinert materials that invoke only a weak‐mild inflammatory response could be advantageous for clinical translation as it removes the “chance” of undesired outcomes and improves the likelihood that the cellular processes are impacted only through the judicious introduction of defined set of cues (mechanical and/or biological). Such cues for instance could range from small molecules to induce macrophage polarization that are immobilized via proteolytically cleavable linkers (for example, sensitive to matrix metalloproteinases (MMPs)), to signals to promote Treg recruitment, signals downstream to Treg recruitment such as sustained IL‐10 or IL‐13 delivery, and motifs to mechanically couple the biomaterial stiffness to invading cells to leverage mechanobiology paradigms. The pros and cons of bioactive versus bioinert materials are summed up in **Table** **2**. Drawing inspiration from biomaterials for immunotherapy,^[^ ^97^ ^]^ we propose that a biological inert background provided by the bioink can preserve the function of signaling peptides or cell binding motifs thus ensuring a highly specialized instructive layer to drive specific cellular processes and outcomes. In this regard, one example of a extrudable hydrogel forming material that is inert and exhibits many of the prerequisites for microextrusion printing including shear thinning, rapid sol–gel transitions and has been explored in 3DBP is carboxylated agarose (CA)^[^ ^56^, ^57^, ^98^ ^]^ (**Figure** **5**). Exploiting a mechanobiology paradigm, it has been shown that CA hydrogels modified with RGD sequence that is necessary for endothelial cell proliferation and with mechanical properties akin to blood clot can promote long‐term stabilization of neo‐vessels. This occurs via the recruitment of circulating monocytes (CD11b^+^/CD115^+^) expressing the stretch‐activated cation channel protein Piezo‐1.^[^ ^4^ ^]^ Since CA hydrogels invoke a weak inflammatory response, they do not undergo a fibrous encapsulation and therefore are permissive to invading host cells and the biological signal presented (i.e., the RGD motif) is effective in driving clear outcomes. These findings provide a strong case for adding bioinert bioinks into the repertoire of bioinks for developing translation 3D‐bioprinted solutions in regenerative therapies. Alginate, another extensively used biopolymer in tissue engineering^[^ ^5^, ^95^ ^]^ has also been widely used in 3DBP^[^ ^58^, ^59^, ^60^ ^]^ and could be a candidate bioink for clinical translation. Alginate has been printed using drop‐on‐demand technique using agarose/gelatin as a support fugitive phase.^[^ ^49^ ^]^ Alginate has been shown to have low‐oral toxicity and skin irritability in animals^[^ ^99^ ^]^ and is currently used in topical wound healing products. However, the safety of alginate for in vivo human use needs to be established and towards this goal, the drawbacks associated with calcium crosslinking need to be overcome, as calcium ions have a role as a secondary messenger in cellular function in concentration‐dependent manner. Extracellular divalent calcium has been recently shown to play a prominent role in the differentiation of fibroblasts into myofibroblasts a key step in fibrotic response.^[^ ^100^ ^]^ Excessive, myoblast activation is the primary driver of pathological fibrosis. Extracellular calcium influx into lung epithelial cells has been implicated in the progression of cystic fibrosis via hyperstimulation of inflammatory processes.^[^ ^101^ ^]^ Marked and sustained elevation in extracellular calcium is implicated in cytoskeletal degradation and apoptosis,^[^ ^102^ ^]^ and high levels of extracellular calcium have been shown to also alter the differentiation potential of human mesenchymal cell and the pathway for bone formation.^[^ ^103^ ^]^

**Table 2:** Pros and cons of bioactive materials and bioinert materials

Type of biomaterials in bioink	Pros	Cons
Bioactive—biomaterials that are derived from multicellular living organisms (e.g., collagen, elastin, hyaluronic acid, silk)	Contain components that can be recognized by human physiology, and processed by cells and endogenous enzymes and proteases	Quality of the biomaterial depends on the tissue source and processing parameters.
	Possess cell adhesion motifs	Can be a potential source of pathogens (viruses)
	Can be degraded in vivo	Unpredictable inflammatory response due to breakdown products (peptides and denatured proteins)
		Limited shelf life
		High cost
		Might involve animal sacrifice
Bioinert—biomaterials that are derived from plants and algae (e.g., agarose, alginate, carrageenan)	Easily sourced, purified and scaled	No cell adhesion motifs, chemical modification with peptides such as RGD necessary to promote cell interaction
	Low cost	Slow degradation through oxidative processes involving macrophages
	Typically, low inflammatory response	
	Absence of inherent biological activity provides a reproducible microenvironment for manipulation of cell function	
	Does not involve animal sacrifice	

## 4 Bioink Development Tool Kit

### 4.1 Rheology

Although rheological characterization has been a routine in development of bioinks, it is still worthwhile to advocate a more comprehensive rheological testing. As already discussed earlier the sterilization process is bound to influence bioink properties, therefore, we propose rheological testing on sterilized bioinks as the basis for clinical translation. Routine tests such as shear rate sweep, temperature sweep, and frequency sweep can provide basic insights into shear property, gelling temperature, and mechanical strength. However, bioprinting is a dynamic process that is also impacted by nozzle size and length, deposition rate, duration of the print, presence of cells and in some instances temperature of the print nozzle. Here, well‐designed rheological tests to simulate these variables can help identify subtle changes in rheology that influence gelation range, cell viability and fusion between printed layers (**Table** **3**). For example, in the case of printing gelatin, temperature‐dependent rheological tests can be used to define the precise temperature at which the gelatin can possess sufficient viscosity due to partial physical crosslinks to support structures while keeping shear stress to the minimum.^[^ ^104^ ^]^ Using similar approaches, the printing temperature for GelMA at various concentrations was optimized.^[^ ^33^ ^]^ One could also offset changes in viscosity and rheological behavior using viscosity modifiers such as gellan and nanosillicates, and here rheology has been found to be predictive of the printability of the bioink.^[^ ^43^, ^105^ ^]^ Although it has been shown in limited studies that low cell content, or cells aggregates have a negligible impact on rheological properties of bioink, cell density is a critical element when printing 3D structures for tissue regeneration. As alluded to earlier, the addition of cells at high density transforms the bioink into a colloidal dispersion which will exhibit different rheological properties compared to non‐cell bioink, potentially impacting gelation kinetics and mechanical properties. For example, it has been reported that at a cell density of 100 million cells mL^–1^ (which accounts for about 30% of the printed volume) the formation of HA‐tetra‐PEG gels via chemical crosslinked was significantly impacted and beyond these concentrations (250 and 500 million mL^–1^) no crosslinking could be achieved.^[^ ^40^ ^]^ Also, it was found that even a modest increase in the concentration of cells from 1.5 to 2.5 million mL^–1^ can significantly lower the gelation temperature of 10% (w/v) GelMA and its shear thinning behavior.^[^ ^106^ ^]^ Therefore, a thorough rheological characterization of cell‐laden bioinks should be carried as a routine to ascertain the performance under bioprinting conditions. In sum, establishing rheological parameters that reflect the intended use of the bioink can provide quantitative information on mechanical forces experienced by cells during the print and further understanding of the impact of the bio‐printing process by itself on cell fate and function.

**Table 3:** Rheological tests to characterize properties of bioinks (Note: All the tests are supposed to performed based on the samples that passed through sterilization. When cells are involved, comparison between non‐cell bioinks and cell‐laden bioinks is recommended)

Rheological tests	Variable	Output
Amplitude sweep	Strain (*γ*)	Storage modulus (*G*’), loss modulus (*G*’’)
Frequency sweep	Frequency (*f*, or *ω*)	*G*’ and *G*’’
Temperature sweep 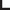 From high temperature (*T*_H_) to low temperature (*T*_L_) or from *T*_L_ to *T*_H_ 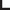 Temperature change during printing	Temperature (*T*)	*G*’ and *G*’’, or viscosity (*η*)
Shear rate sweep	Shear rate (*γ̇*)	*G*’ and *G*’’, or *η*
Time sweep 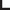 At holding temperature	Time (*t*)	*G*’ and *G*’’, or *η*
Yield stress 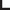 Simulating bioink passing through pipes with different diameters	*γ̇*	Shear stress (*σ*)
Strain–stress (compressive)	Compressive strain (*γ*)	Normal force (N)

### 4.2 Crosslinking

In extrusion printing, the transient physical shape prescribed to the bioink up on extrusion from the nozzle has to stabilized to yield an object that can withstand handling, and culturing. This is typically achieved through physical, ionic, or covalent crosslinking. A compendium of the most common crosslinking methodologies is listed in **Table** **4**. In several studies, structures were first printed into temporarily stable structures (with weak physical crosslinking), and then postprocessed by UV light‐induced chemical crosslinking to form stable structures.^[^ ^33^, ^42^ ^]^ However, with deeper understanding of polymer physics during printing, and development of new biomaterials that can be processed during the extrusion of the bioink, crosslinking is no longer a single‐phase effector but a complex, multiphase process, to build higher and more complex hierarchical structures. For example, in situ crosslinking can work as viscosity enhancer and stabilize the structure for further processing. Using a coaxial nozzle delivering calcium solution in centra nozzle, rapid gelation of alginate was achieved enabling preservation of the printed geometry during the print.^[^ ^107^ ^]^ As stated earlier, physical supports can also serve to stabilize structures during printing. In FRESH printing process, real‐time crosslinking by divalent cations (Ca^2+^) was used to preserve the structures in addition to temporary physical support from the gelatin slurry.^[^ ^48^ ^]^ However, systems that do not require a physical support medium during print or postprocessing to print physically stable structures can accelerate the regulatory process and favor clinical translation. Here, physically gelling systems, most notably, CA‐based bioinks offer clear advantages, namely, shear thinning, decoupling of solution viscosity from stiffness of printed structures, capability to yield complex structures at physiological conditions, and more importantly, ability to promote stable angiogenesis without any fibrotic or inflammatory response.^[^ ^4^, ^56^, ^57^, ^98^ ^]^ Nonetheless, there is a clear role for strategies to improve printability of biological compatible materials. From the above‐mentioned studies, it is evident that crosslinking strategies in 3DBP are not simply a post‐printing processing step but also as an enabler and fluent link between the three phases of printing (extrusion, temporary, and long‐term preservation of geometry). Thus, identifying functionalization and crosslinking chemistries that are reproducible and independent of user skill‐level is pivotal (i.e., a biologist can equally implement with the same efficiency as a materials scientist). From a translational standpoint, atom‐economy reactions such as (Diels‐Alder, inverse electron demand Diels‐Alder, 1,3 dipolar cycloadditions “click chemistry”, thiol–ene) are ideal as they do not generate byproducts and are also amenable to in line processing of the bioink.

**Table 4:** Molecular interactions/chemistries and physicochemical properties in the next generation bioinks and their implications for 3D bioprinting and clinical translation

Molecular interactions/chemistries	Physicochemical properties	Implications for printing, translation, signaling paradigm
Click chemistry	Covalent crosslinking	Postprinting stabilization of print
Helical‐helical, *β*‐sheet‐strand, helical‐*β*‐sheet	Physical crosslinking, thermal gelation, hydrocolloid network	Printing at physiological and room temperature, graded gelation, free standing structures, printing gels, printing high cell concentration 10–100 million cells per cc
Hydrophobic (lipid–lipid, cholesterol derived structures, liquid crystal moieties)	LCST^a)^ behavior, shear thinning,	Degradable fugitive phase for de novo evolution of controlled macro architecture for vascularization and innervation.
Host–guest (cyclodextrins), Schiff's base	Shear thinning	Room temperature printing
Peptide–peptide, peptide–polysaccharide, aptamer–peptide, light sensitive proteins (rhodopsin)	Multi network (double, triple) hydrogels, active cellular remodeling, potentially shear‐thinning, room temperature gelation, pH‐triggered gelation and disassembly, super elastic scaffolds,	Modulation of viscosity and biofunctionality during print (in line processing), biofunctionalization, extreme customization
Light sensitive moieties (azo benzene) and proteins	Light activated networks	Mechanobiology, light responsive systems, systems responsive optogenetics
Light activated cross linking (thiol‐e(y)ne, tyrosine‐tyrosine) and thermally activated systems as Diels‐Alder, inverse electron donor Diels‐Alder		In‐line processing using light, modulating viscosity during print, real‐time stabilization of printed structures, biofunctionalization during print, extreme customization of biology

^a)^ LCST = lower critical solution temperature.

### 4.3 Potential Toolkit in In Vitro and In Vivo Models: Next‐Generation Sequencing and Epigenetics

In 3DBP involving cells, cell viability is an important and fundamental evaluation index for printing efficiency, and most studies on 3DBP have reported this value. It is understandable that majority of studies put a lot of emphasis on elevating postprinting cell viability in the initial developing stage of 3DBP, as in this stage plenty of studies on printable biomaterials and hardware have reported in which simple and basic biological data were deemed quite adequate to catch the “readers’ attention.” However, real advances could only be propelled by in‐depth and specific characterizations of the output of 3DBP that emphasizes biological functional attributes and correlation to in vivo outcomes. Many published studies will not pass this stringent filter. These specific characterizations include clinically relatable in vitro studies, and ectopic and orthotopic in vivo models to verify end points. Maintaining the cells’ phenotype and functions is the prerequisite for cross‐platform (in vitro through in vivo) validation. Whether 3DBP, the postprinting environment, and biomaterials per se would influence cells’ functions could be investigated at different levels, namely, characteristic cell marker proteins, analysis of lineage and function‐related genes using transcriptomics to identify differential gene expression, epigenetic changes using DNA methylation, alternative splicing, single cell proteomics, and whole genome sequencing. Such in‐depth characterization is routine in biology, and gaining traction in tissue engineering with bone and cartilage being the most studied engineered tissue^[^ ^17^ ^]^, and this needs to be explicitly incorporated in 3DBP studies. However, so far there have been few studies on how biomaterials and 3DBP process affects cell phenotype. The role of material cues in regulating epigenetics and cell function has become a noteworthy topic. Materials can serve as a medium to investigate mechanobiology paradigms in cells. The nucleus can sense the mechanical cues from extracellular environment (e.g., cells, ECM) either physically through linker of nucleoskeleton and cytoskeleton complex (LINC) that is responsible for force propagation^[^ ^108^ ^]^ or via mechano‐gated ion channels, such as TREK/TRAAK K_2P_ channels, Piezo1/2, TMEM63/OSCA, and TMC1/2.^[^ ^8^, ^109^ ^]^ For example, Blumenthal et al., showed that stochastic nanoroughness, a means to impart mechanical cues to cell membrane, can regulate the interaction of astrocytes and hippocampal neurons via the mechanosensing protein Piezo‐1 and also dictate telencephalic neural progenitor cell fate choices.^[^ ^8^ ^]^ Furthermore, in cancer progression matrix stiffness has been shown to correlate to negative prognosis.^[^ ^110^ ^]^ Whether the anisotropic mechanical forces exerted on cells during 3DBP, which include shear stress during extrusion, stiffness change during gelation or crosslinking, and anisotropic inner forces inside complex structures, can activate mechanotransduction paradigms in cells after printing, leading to mRNA changes, DNA methylation, or post‐translational histone modifications^[^ ^111^ ^]^ need further investigation. Additionally, whether the micro or nano‐scale cues influence cell fate and behavior in histogenesis, and organogenesis remains an interesting topic for further study. With the increased access and reduced cost of the next‐generation sequencing (NGS) and tools to study epigenetics, they could become routine and effective tools to compare the genomes of cells after 3DBP. Such data could be valuable in establishing valid and realistic models of organs and physiological processes and diseases such as tumors.

## 5 Summary

In summary, the evolution of 3DBP into a bona fide clinical technology hinges on the ability to demonstrate clinical feasibility. Bioinks have a critical role in driving this transformation. In the development of translatable bioinks, many strategies need to be married to meet the desired property profile, and the proposed tool kit can be used to adjust the formulation and optimize the fabrication process. Here we advocate a rigorous approach to identify bioinks, and a biofabrication process that has fewer variables and moving parts to promote efficiency, reproducibility, and a streamlined translation process; and more importantly places the clinical end point (or use) front and center. However, whether the bioink can enter the clinic still depends on strict preclinical evaluation. While, certainly not all the bioinks can satisfy the criteria necessary for translation into clinics, bioinks that do satisfy the use criteria for TRL3 and TRL4 can still be exploited as platform in building in vitro model for drug screening and studying signaling and crosstalk in complex cellular environments and serve as test beds for translation. Those bioinks that enter TRL4 but fail to enter TRL5 can still serve as a platform for building in vitro patient‐specific disease models that can be further investigated in disease‐specific animal models. While there has been tremendous progress in the development of bioinks in the past five years, innovations in bioprinting platform have lagged. There is a need to drive a convergence between properties of bioinks with high clinical potential and bioprinter workflow and specifications in order to maximize successful clinical outcomes. It is anticipated that this perspective will spur an accelerated development of a standardization matrix and clinically translatable bioinks.

## Conflict of Interest

The authors declare no conflict of interest.
